# Patient-reported experience with Fabry disease and its management in the real-world setting: results from a double-blind, cross-sectional survey of 280 respondents

**DOI:** 10.1186/s13023-024-03090-4

**Published:** 2024-04-11

**Authors:** Lisa Berry, Jerry Walter, Jack Johnson, Julia Alton, Janet Powers, Xavier Llòria, Irene Koulinska, Meghan McGee, Dawn Laney

**Affiliations:** 1 Cincinnati Children’s Hospital Medical Center, Cincinnati, OH USA; 2 National Fabry Disease Foundation, Hillsborough, NC USA; 3 Fabry Support & Information Group, Concordia, MO USA; 4 Canadian Fabry Association, Thunder Bay, ON Canada; 5 MedPanel, Inc., Waltham, MA USA; 6 Chiesi Global Rare Diseases, Parma, Italy; 7 Chiesi USA, Inc., Boston, MA USA; 8 Emory University School of Medicine, Atlanta, GA USA

**Keywords:** Fabry disease, Patient experience, Enzyme replacement therapy, Chaperone therapy, Patient-reported outcome, Rare disease

## Abstract

**Background:**

Fabry disease (FD) is a rare X-linked lysosomal storage disorder with a heterogeneous clinical presentation. Patients with FD may exhibit early signs/symptoms including neuropathic pain, gastrointestinal complaints, and dermatologic manifestations. FD may ultimately progress to renal, neurologic, and cardiac dysfunction. Current treatments for FD have significantly improved the management and outcomes for patients with FD, but important clinical and convenience limitations still exist.

**Methods:**

To illuminate the impact of FD on daily life from the patient’s perspective, we asked adult patients (≥ 18 years old) with FD in the United States and Canada to complete a 33-question online survey to assess patient-reported disease severity, management, and treatment outcomes.

**Results:**

A total of 280 respondents with FD completed the survey; they had a mean age of 47 years, and 68% (191/280) were women. Most were currently receiving FD treatment (84%, 234/280) with enzyme replacement therapy (ERT) (89%, 208/234) or chaperone therapy (11%, 26/234). Common symptoms included low energy/fatigue (72%, 201/280), tingling (62%, 174/280) or pain in the hands/feet (60%, 168/280), ringing in ears/hearing loss (54%, 151/280), general body pains/pain crises (51%, 143/280), and abdominal/stomach pain (50%, 140/280). More than half (51%, 144/280) of respondents reported their symptoms as bothersome (38%, 106/280) or difficult to control (14%, 38/280). Temporary symptom worsening between infusions was reported by about half of respondents: 51% (108/211) currently receiving ERT and 48% (14/29) previously receiving ERT. Only 48% (59/122) of respondents reported their symptom worsening to their physician. Of those who reported it, 41% (24/59) said that their physician prescribed medication to manage their symptoms or changed their treatment regimen.

**Conclusions:**

Our analysis highlights the gap between current standard-of-care in disease monitoring and patient perception of disease progression among patients with FD. This information may be helpful for healthcare providers and drug developers seeking to improve the care of patients with FD by addressing unmet needs of high relevance.

**Supplementary Information:**

The online version contains supplementary material available at 10.1186/s13023-024-03090-4.

## Background

Fabry disease (FD; OMIM 301,500) is a rare X-linked lysosomal storage disorder caused by pathogenic variants in the *GLA* gene resulting in α-galactosidase A deficiency and cellular accumulation of globotriaosylceramide and related glycosphingolipids [1–3]. Patients with FD exhibit heterogeneity in their clinical presentation and disease course, falling on a disease spectrum that ranges from a classic, severe phenotype that manifests during childhood or adolescence to a nonclassic, milder phenotype with a later onset [2, 4]. Depending on phenotype, patients may experience progressive dysfunction of multiple organ systems, with early signs and symptoms in childhood that include neuropathic pain, gastrointestinal (GI) complaints (e.g. diarrhoea, chronic constipation, and/or abdominal pain), and dermatologic manifestations (e.g. angiokeratomas) [1, 2]. As the disease progresses, patients may then experience deterioration of renal, neurologic, and cardiac functions [1, 2]. As such, patients require an individualized therapeutic approach that includes promptly initiating treatment and symptom management [5].

Currently, there are three approved treatments for FD in Canada: two enzyme replacement therapies (ERT), agalsidase beta (Fabrazyme) and agalsidase alfa (Replagal), and one oral chaperone therapy, migalastat (Galafold) [6–8]. Agalsidase beta and migalastat are also approved in the United States, and a new ERT, pegunigalsidase alfa-iwxj (Elfabrio), was approved in May 2023 after the present study was completed [9–11]. ERT and chaperone therapy have significantly improved the management of patients with FD; however, both therapies carry important clinical and convenience limitations that in some cases may affect long-term clinical outcomes [12, 13]. ERT is administered by intravenous infusion every 2 weeks and can be associated with the development of anti-drug antibodies (ADAs) and infusion-related reactions (IRRs) [6, 8, 9]. In clinical trials, ADAs were reported in 83% of adult patients treated with agalsidase beta and 9.4% of male patients treated with agalsidase alfa, and IRRs were reported in 59% of patients treated with agalsidase beta and 13.7% treated with agalsidase alfa [8, 9]. The occurrence of ADAs and IRRs may necessitate premedication and prolonged infusion times [2, 14], which may pose a heavy burden for patients.

Another challenge identified with currently approved ERTs is the short plasma half-life [15] that may result in low functional enzyme levels during the second week of a 2-week dosing regimen [16], which may contribute to patient reports of symptom worsening between infusions. Additionally, although chaperone therapy provides a more convenient oral route of administration and is not subject to ADA development, its usage is limited to a subset of patients that carry specific amenable mutations, which are present in approximately 35–50% of patients with FD [13, 17].

To better understand the challenges associated with treatment and its impact on daily living, researchers may solicit feedback directly from patients with FD [18]. Patient-reported outcomes (PROs), which are reports of a patient’s health condition conveyed directly by the patient without interpretation by the patient’s healthcare provider or any other person, are tools that are often used for this purpose [18, 19]. Examples of PROs include disease symptoms or treatment side effects such as pain, fatigue, or anxiety; functional outcomes such as physical, sexual, social, emotional, or cognitive functioning; or multidimensional constructs such as health-related QoL or health utility [20, 21]. However, PROs have not historically been included as primary endpoints in clinical trials. To date, only a few real-world evidence (RWE) studies of patients with FD have been published [22–24]. In an international online survey of patients with FD, similar proportions of patients reported moderate to severe pain, whether they were receiving ERT (80.4%, 225/280 patients) or not (75.0%, 63/84) [22]. In an online survey of Japanese patients with FD and their treating physicians, only about half of patients (53.3%, 16/30) thought their FD symptoms were manageable with hospital visits and treatment, whereas two-thirds of their matched treating physicians (66.7%, 20/30) thought their patients’ symptoms were manageable. Physicians also placed greater emphasis on laboratory values (e.g. cardiac and renal values) than disease symptoms (e.g. GI complaints and sweating abnormalities) when considering the impact of FD on patient QoL [23]. In another survey of Japanese patients with FD currently being treated with ERT, more than half of patients reported ongoing symptoms of FD while being treated with ERTs, including cardiac function-related manifestations (63%), fatigue (58%), limb pain (55%), and neurologic manifestations (53%) [24]. Considered together, these studies suggest that patients with FD may continue to experience symptoms despite treatment [22–24].

Given the paucity of published data related to the patient’s perspective of their FD, collecting additional RWE from this patient population may provide valuable insights about the management and monitoring of patients with FD for healthcare providers, researchers, industry partners, and to those affected by the disease [18, 25]. Therefore, in the present analysis, we sought to assess patient perception of FD progression, severity, and management, as well as patient satisfaction with monitoring and treatment.

## Methods

### Design and development

The survey was developed with input on content and question wording from representatives from two FD patient advocacy groups, two genetic counsellors, and a geneticist experienced with FD. A pilot ‘soft launch’ of the questionnaire was conducted with fewer than 10 participants, followed by 60-minute, web-assisted, telephone cognitive interviews with two participants to ensure survey questions were clear and correctly interpreted by respondents. The language of survey questions and answer options were then revised based on feedback from participants during the soft launch.

The survey was conducted with the ethical principles that have their origins in the Declaration of Helsinki, in compliance with the approved protocol, Guidance on Good Clinical Practice guidelines, and applicable regulatory requirements. Approval was obtained from Western Institutional Review Board, now known as WIRB-Copernicus Group, before enrolling any participants. All participants provided written informed consent.

The survey was administered by MedPanel (Chatham, MA, USA), a third-party healthcare market research firm experienced in rare diseases. The finalized 30-minute online survey was double-blind, meaning that participants were unaware of who sponsored the survey, and all data were deidentified before being provided to the sponsor. Survey responses were collected over 15 days starting on 2 February 2022 from participants with FD in the United States and Canada.

**Fig. 1:**
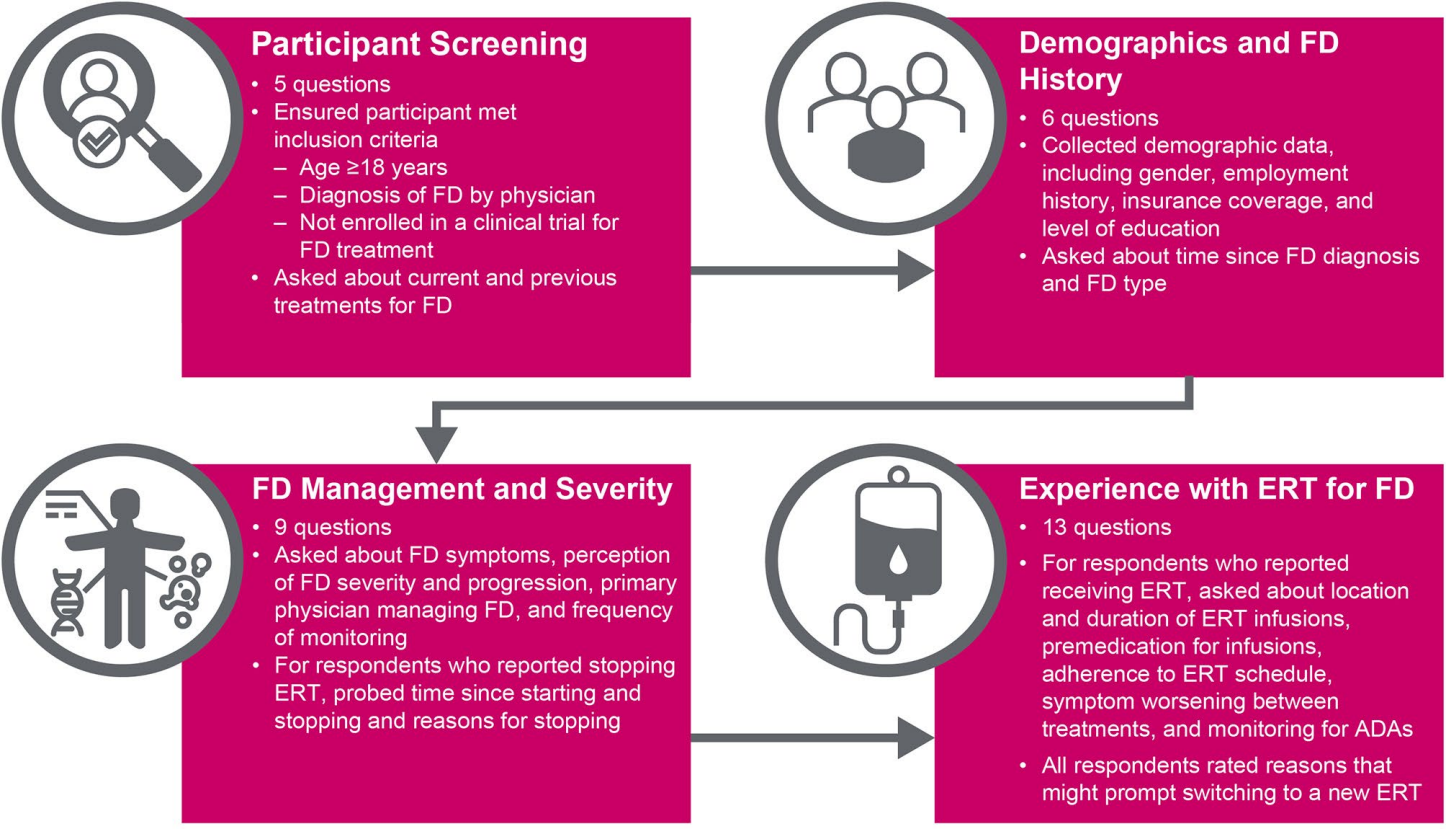
Overview of the Fabry Disease Survey. ADA, antidrug antibodies; ERT, enzyme replacement therapy; FD, Fabry disease

The final survey consisted of five screening questions and 28 survey questions (Fig. 1 and Additional file 1: Table S1). The first three screening questions ensured that patients met inclusion criteria, and the other two screening questions collected data on previous and current treatments for FD. The 28 survey questions covered three primary areas: demographics and FD history, FD management and severity, and experiences with ERT for FD. The questions pertaining to each of these areas were chosen with the overall goal of assessing patterns of disease monitoring, understanding the patient’s perception of the burden of disease, and learning about the impact of therapy on patients’ QoL. Most questions were yes/no or multiple choice, and three questions asked participants to select their answer on a 5-point Likert scale. The following are examples of questions from the survey:

- How would you characterize the severity of your Fabry disease symptoms? Multiple choice: mild-moderate symptoms; bothersome symptoms; symptoms are difficult to control.
- How often do you feel that your disease might be worsening even if all the labs and assessments during your doctor(s) visit(s) appear stable? Multiple choice: never; sometimes; often.
- Do you experience temporary worsening of symptoms in the days between ERT infusions? Yes; no.

### Participants

Patient advocacy groups and physician and patient referrals were used to recruit participants. To be included in the survey, participants had to be at least 18 years old, understand English, and have a diagnosis of FD from a healthcare professional, which MedPanel confirmed via patient-provided documentation. Participants could not be enrolled in a clinical trial for FD treatment at the time of the survey. All participants were compensated for their time.

### Analysis

Participant responses were deidentified before analysis by the sponsor. Descriptive data are presented for each question as absolute numbers (n) and percentages. Data were stratified by gender (omitting report of non-conforming gender selection due to low response number [*n* = 1]), age, and treatment status to identify trends, and subgroup comparisons are reported where relevant or where different trends were noted. Because the data collected were qualitative in nature, statistical tests were not performed.

## Results

### Demographics and disease history

A total of 280 participants with FD completed the survey (Table 1). Respondents ranged in age from 18 to 77 years, with a mean age of 47 years. The majority of participants were female (68%, 191/280; male: 31%, 88/280; non-conforming: 1%, 1/280). Most respondents reported they were currently receiving FD treatment (84%, 234/280; male: 95%, 84/88; female: 78%, 149/191); the remainder were not currently receiving any treatment (13%, 37/280; male: 1%, 1/88; female: 19%, 36/191), or they selected “other” (3%, 9/280; male: 3%, 3/88; female: 3%, 6/191) for treatment status. Of those currently receiving treatment for FD, 89% (208/234) were receiving ERT with agalsidase beta (91%, 189/208) or agalsidase alfa (9%, 19/208), and 11% (26/234) were receiving chaperone therapy with migalastat. Respondents not currently receiving any treatment were either treatment naïve (10%, 28/280) or had previously been on ERT but had discontinued (3%, 9/280). Examples of free text responses for “other” included “infusion,” “PRX-102” (pegunigalsidase alfa), and “preparing to begin treatment.”

**Table 1:** Demographic characteristics and disease history, stratified by treatment status

Characteristic, n (%)	All (*N* = 280)	Currently treated			Previously treated with ERT (*n* = 9)	Treatment naïve (*n* = 28)	Other (*n* = 9)
		Agalsidase beta (*n* = 189)	Agalsidase alfa (*n* = 19)	Migalastat (*n* = 26)			
Gender							
Female	191 (68)	120 (63)	10 (53)	19 (73)	9 (100)	27 (96)	6 (67)
Male	88 (31)	68 (36)	9 (47)	7 (27)	0 (0)	1 (4)	3 (33)
Non-conforming	1 (0)	1 (1)	0 (0)	0 (0)	0 (0)	0 (0)	0 (0)
Age group							
18−44 years	124 (44)	82 (43)	12 (63)	9 (35)	5 (56)	11 (39)	5 (56)
45−64 years	118 (42)	82 (43)	6 (32)	9 (35)	3 (33)	14 (50)	4 (44)
≥65 years	38 (14)	25 (13)	1 (5)	8 (31)	1 (11)	3 (11)	0 (0)
Education level							
Received masters or above	52 (19)	32 (17)	1 (5)	6 (23)	2 (22)	9 (32)	2 (22)
Finished college	106 (38)	77 (41)	3 (16)	11 (42)	3 (33)	8 (29)	4 (44)
Finished trade school	27 (10)	19 (10)	4 (21)	1 (4)	1 (11)	1 (4)	1 (11)
Finished high school/received GED	88 (31)	56 (30)	11 (58)	8 (31)	3 (33)	9 (32)	1 (11)
Did not finish high school	7 (3)	5 (3)	0 (0)	0 (0)	0 (0)	1 (4)	1 (11)
Employment status							
Employed full- or part-time	152 (54)	103 (54)	11 (58)	11 (42)	3 (33)	20 (71)	4 (44)
Retired	82 (29)	57 (30)	3 (16)	13 (50)	4 (44)	2 (7)	3 (33)
Not employed or retired	17 (6)	10 (5)	3 (16)	0 (0)	1 (11)	2 (7)	1 (11)
Student full- or part-time	15 (5)	11 (6)	2 (11)	1 (4)	0 (0)	1 (4)	0 (0)
Stay-at-home household manager	14 (5)	8 (4)	0 (0)	1 (4)	1 (11)	3 (11)	1 (11)
Health insurance							
Commercial/private	157 (56)	108 (57)	10 (53)	12 (46)	5 (56)	18 64)	4 (44)
Medicare	71 (25)	49 (26)	3 (16)	8 (31)	5 (56)	3 (11)	3 (33)
Medicaid	29 (10)	19 (10)	4 (21)	3 (12)	2 (22)	0 (0)	1 (11)
Marketplace	12 (4)	9 (5)	1 (5)	2 (8)	0 (0)	0 (0)	0 (0)
Military	8 (3)	6 (3)	0 (0)	1 (4)	0 (0)	1 (4)	0 (0)
Other	27 (10)	19 (10)	1 (5)	2 (8)	1 (11)	4 (14)	0 (0)
Not insured	4 (1)	1 (1)	0 (0)	0 (0)	0 (0)	2 (7)	1 (11)
Classic FD							
Yes	181 (65)	136 (72)	13 (68)	10 (38)	6 (67)	9 (32)	7 (78)
No	49 (18)	19 (10)	5 (26)	11 (42)	2 (22)	11 (39)	1 (11)
Not sure	50 (18)	34 (18)	1 (5)	5 (19)	1 (11)	8 (29)	1 (11)
Time since FD diagnosis							
<1 year	5 (2)	0 (0)	0 (0)	0 (0)	0 (0)	4 (14)	1 (11)
1−2 years	24 (9)	15 (8)	6 (32)	2 (8)	0 (0)	1 (4)	0 (0)
3−5 years	58 (21)	34 (18)	8 (42)	7 (27)	1 (11)	7 (25)	1 (11)
6−10 years	49 (18)	37 (20)	1 (5)	5 (19)	2 (22)	4 (14)	0 (0)
>10 years	144 (51)	103 (54)	4 (21)	12 (46)	6 (67)	12 (43)	7 (78)
Physician managing FD							
Geneticist	128 (46)	90 (48)	3 (16)	15 (58)	3 (33)	13 (46)	4 (44)
Nephrologist	63 (23)	48 (25)	5 (26)	2 (8)	3 (33)	4 (14)	1 (11)
Primary care/family doctor	45 (16)	27 (14)	5 (26)	5 (19)	2 (22)	3 (11)	3 (33)
Cardiologist	20 (7)	12 (6)	5 (26)	2 (8)	1 (11)	0 (0)	0 (0)
Other	10 (4)	7 (4)	0 (0)	1 (4)	0 (0)	2 (7)	0 (0)
Neurologist	8 (3)	5 (3)	1 (5)	1 (4)	0 (0)	1 (4)	0 (0)
No one	6 (2)	0 (0)	0 (0)	0 (0)	0 (0)	5 (18)	1 (11)

ERT, enzyme replacement therapy; FD, Fabry disease; GED, General Educational Development

Respondents were highly educated, with most (56%, 158/280) reporting that they had completed college (38%, 106/280) or a master’s degree or above (19%, 52/280). More than half (54%, 152/280; male: 65%, 57/88; female: 50%, 95/191) of respondents were employed full-time or part-time. Nearly one-third (29%, 82/280) reported being retired; of those, 61% (50/82) indicated that this was due to disability and their average age was 54 years (25–70). All respondents who reported themselves to be stay-at-home household managers were female (*n* = 14). Most respondents had health insurance, with more than half (56%, 157/280) reporting they had commercial or private insurance and more than one-third (36%, 100/280) reporting they had government insurance from Medicare or Medicaid. A total of 4 respondents of 280 (1%) reported having no insurance and were all ≤ 64 years; 3% (3/88) of males and less than 1% (1/191) of females were not insured.

Approximately half of respondents were diagnosed with FD more than 10 years ago (51%, 144/280). Nearly two-thirds (65%, 181/280; males: 70%, 62/88; females: 62%, 118/191) reported that they had been diagnosed with classic FD. Notably, respondents with classic FD comprised 68% (159/234) of those currently receiving treatment.

### Disease management and severity

Respondents most often reported that their FD was primarily managed by a geneticist (46%, 128/280). Other managing physicians included a nephrologist (23%, 63/280), a primary care or family physician (16%, 45/280), a cardiologist (7%, 20/280), and a neurologist (3%, 8/280). Six respondents (2%, 6/280) reported that they did not have a physician managing their FD. All respondents currently (*n* = 208) or previously receiving ERT (*n* = 9) reported having their disease managed by a physician, whereas nearly one-fifth (18%, 5/28) of treatment-naïve respondents did not have a physician managing their disease. Respondents who were currently receiving agalsidase beta (48%, 90/189) or chaperone therapy (58%, 15/26), as well as those who were treatment naïve (46%, 13/28) were most often managed by a geneticist, whereas those currently receiving agalsidase alfa were most often managed by a nephrologist, a primary care or family physician, or a cardiologist (each 26%, 5/19).

**Fig. 2:**
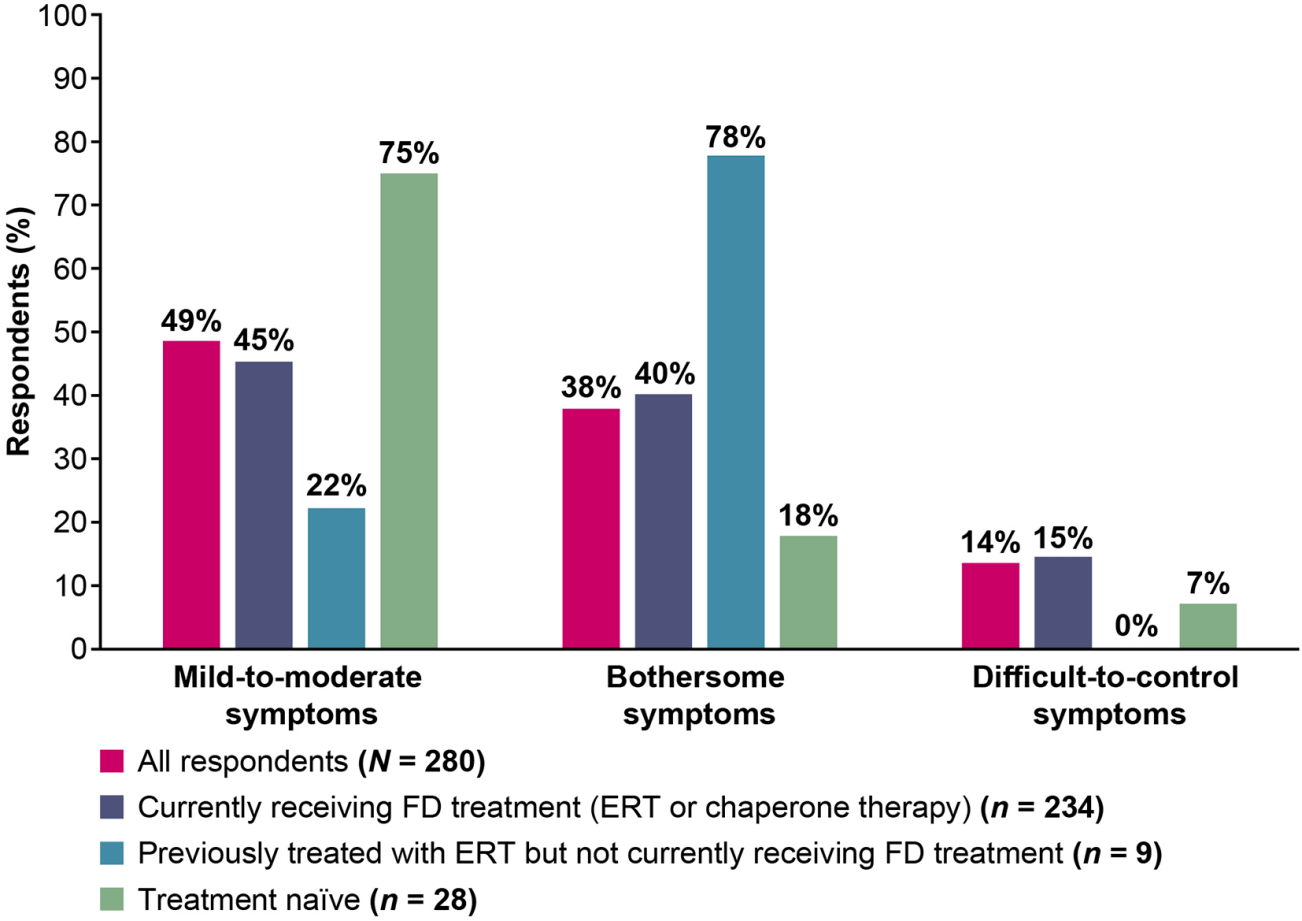
Fabry disease symptom severity. ERT, enzyme replacement therapy; FD, Fabry disease

The questionnaire delineated the proportion of respondents who experienced various symptoms of FD, and these are reported in Table 2. The most common symptoms overall were low energy or fatigue (72%, 201/280), tingling (62%, 174/280) or pain in the hands and/or feet (60%, 168/280), ringing in ears and/or hearing loss (54%, 151/280), general body pains and/or pain crises (51%, 143/280), and abdominal and/or stomach pain (50%, 140/280). Of the common symptoms, 73% (90/124) of those aged 18–44 years reported tingling in extremities, whereas low energy/fatigue was most commonly reported by those aged above 45 years (78%, 122/156); there were no clear differences when stratified by gender. When asked to characterize the severity of their FD symptoms, nearly half reported symptoms were mild-to-moderate (49%, 136/280), and the remainder reported they were bothersome (38%, 106/280) or difficult to control (14%, 38/280). Compared to the overall population, symptom severity was similar for those currently receiving treatment (Fig. 2). A larger percentage of treatment-naïve respondents (75%, 21/28) reported mild-to-moderate symptoms compared with those currently receiving treatment (45%, 106/234). When considering gender, males equally reported having bothersome or mild-moderate symptoms (41% each, 36/88), while more females reported their symptoms as mild-moderate (52%, 99/191) than bothersome (37%, 70/191).

**Table 2:** Symptoms of Fabry disease probed by the survey

Symptom, n (%)	All (*N* = 280)	Currently treated			Previously treated with ERT (*n* = 9)	Treatment naïve (*n* = 28)	Other (*n* = 9)
		Agalsidase beta (*n* = 189)	Agalsidase alfa (*n* = 19)	Migalastat (*n* = 26)			
Low energy/fatigue	201 (72)	143 (76)	9 (47)	18 (69)	9 (100)	15 (54)	7 (78)
Tingling in hands or feet	174 (62)	115 (61)	11 (58)	19 (73)	7 (78)	15 (54)	7 (78)
Pain in hands or feet	168 (60)	119 (63)	4 (21)	18 (69)	8 (89)	12 (43)	7 (78)
Ringing in the ears or hearing loss	151 (54)	109 (58)	4 (21)	17 (65)	4 (44)	11 (39)	6 (67)
General body pains/pain crises	143 (51)	102 (54)	6 (32)	11 (42)	7 (78)	10 (36)	7 (78)
Abdominal/stomach pain	140 (50)	97 (51)	5 (26)	9 (35)	7 (78)	15 (54)	7 (78)
Brain fog	137 (49)	101 (53)	6 (32)	9 (35)	7 (78)	10 (36)	4 (44)
Skin problems (e.g., angiokeratomas, dry skin)	132 (47)	89 (47)	6 (32)	15 (58)	6 (67)	12 (43)	4 (44)
Sleep disturbances	125 (45)	89 (47)	6 (32)	15 (58)	4 (44)	7 (25)	4 (44)
Anxiety	118 (42)	80 (42)	7 (37)	10 (38)	5 (56)	11 (39)	5 (56)
Other stomach issues (e.g., nausea, vomiting, constipation)	113 (40)	74 (39)	8 (42)	10 (38)	4 (44)	12 (43)	5 (56)
Diarrhoea	112 (40)	73 (39)	8 (42)	10 (38)	3 (33)	12 (43)	6 (67)
Disturbed sweating (reduced or increased)	111 (40)	77 (41)	7 (37)	8 (31)	5 (56)	8 (29)	6 (67)
Depression	108 (39)	73 (39)	8 (42)	11 (42)	4 (44)	6 (21)	6 (67)
Headaches/migraines	106 (38)	74 (39)	8 (42)	11 (42)	5 (56)	5 (18)	3 (33)
Palpitations or chest pain	106 (38)	80 (42)	4 (21)	9 (35)	7 (78)	5 (18)	1 (11)
Shortness of breath	104 (37)	77 (41)	4 (21)	10 (38)	6 (67)	4 (14)	3 (33)
Vision problems	94 (34)	60 (32)	5 (26)	8 (31)	9 (100)	8 (29)	4 (44)
No desire to engage in social activities	68 (24)	50 (26)	3 (16)	6 (23)	3 (33)	2 (7)	4 (44)
Other^a^	18 (6)	12 (6)	0 (0)	2 (8)	1 (11)	3 (11)	0 (0)

^a^Examples of responses for other included coughing, vertigo, balance issues, and numbness in hands or feet

**Fig. 3:**
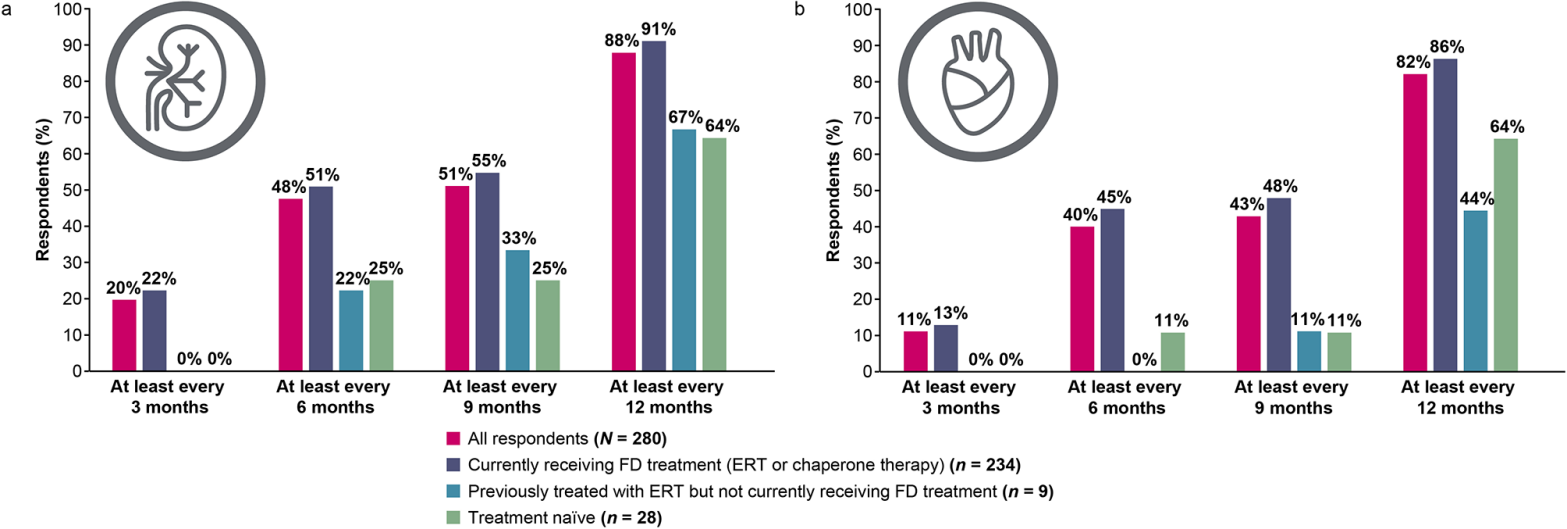
Monitoring frequency for kidney and heart function. (**A**) Kidney function. (**B**) Heart function. ERT, enzyme replacement therapy; FD, Fabry disease

Respondents were asked to indicate how often their kidney or heart function was monitored by laboratory tests or examinations (Fig. 3). Overall, 48% (133/280) had their kidney function monitored at least every 6 months, and 88% (246/280) had their kidney function monitored at least every 12 months. Similarly, 40% (112/280) had their heart function monitored at least every 6 months, and 82% (230/280) had their heart function monitored at least every 12 months. When stratified by treatment status, a higher proportion of those currently receiving treatment were monitored at every time point compared with those who had previously received ERT but had discontinued it or those who were treatment naïve. In consideration of gender, there were more males (35%, 31/88) receiving laboratory testing for assessment of kidney function every 3 months than females (13%, 24/191); females were more likely to be assessed once a year (41%; 79/191 vs. 27%, 24/88). A similar trend was observed for heart function evaluations.

More than half of all respondents reported stable disease without major kidney or heart damage (53%, 147/280). Among those currently receiving treatment, nearly half (48%, 112/234) reported stable disease; the remainder reported that their kidney or heart function was already affected (38%, 90/234) or that they had worsening kidney or heart function damage (14%, 32/234). Among those not currently receiving treatment, most reported stable disease (78%, 29/37). When stratified by gender, females were more likely to report stable disease (58%, 110/191) than males (41%, 36/88); males (24%, 21/88) were more likely to report worsening kidney or heart function damage than females (7%, 13/191). Over 60% (80/124) of patients aged 18–44 years reported stable disease, compared with 48% (57/118) and 26% (10/38) of patients aged 45–64 and over 65 years, respectively.

Most respondents (82%, 230/280) were satisfied with how their disease progression was being monitored, with 36% (102/280) reporting that their disease was monitored “well” and 23% each reporting that it was monitored “moderately well” (65/280) or “excellent” (63/280). A larger percentage of respondents currently receiving treatment expressed a positive perception of monitoring of disease progression compared with those who had previously been treated or who were treatment naïve. Although most respondents were satisfied with their disease monitoring, most (82%, 230/280) also perceived their disease as worsening even when laboratory tests and clinical assessments appeared stable.

### Experience with ERT

Three-quarters of respondents (75%, 211/280) reported currently receiving ERT at the time of completing the questionnaire, and the remainder had been on ERT but discontinued (10%, 29/280) or had never tried ERT (14%, 40/280). Women comprised a greater proportion of those who had never tried ERT (90%, 36/40) or had tried ERT but discontinued (79%, 23/29) than those who were currently receiving ERT (63%, 132/211). Among respondents who reported currently receiving ERT, 30% (64/211) started treatment more than 10 years prior, 22% (46/211) started 6 to 10 years prior, and 27% (56/211) started 3 to 5 years prior. Among those who reported that they had previously been on ERT but had discontinued, the most common probed reason for discontinuation was switching to oral treatment (52%, 15/29); other probed reasons included lengthy infusions, IRRs, and insurance issues (each 14%, 4/29); additionally, four spontaneously reported COVID-19 as another reason for discontinuation. Respondents were able to choose multiple reasons if more than one applied.

### Burden of ERT administration

**Fig. 4:**
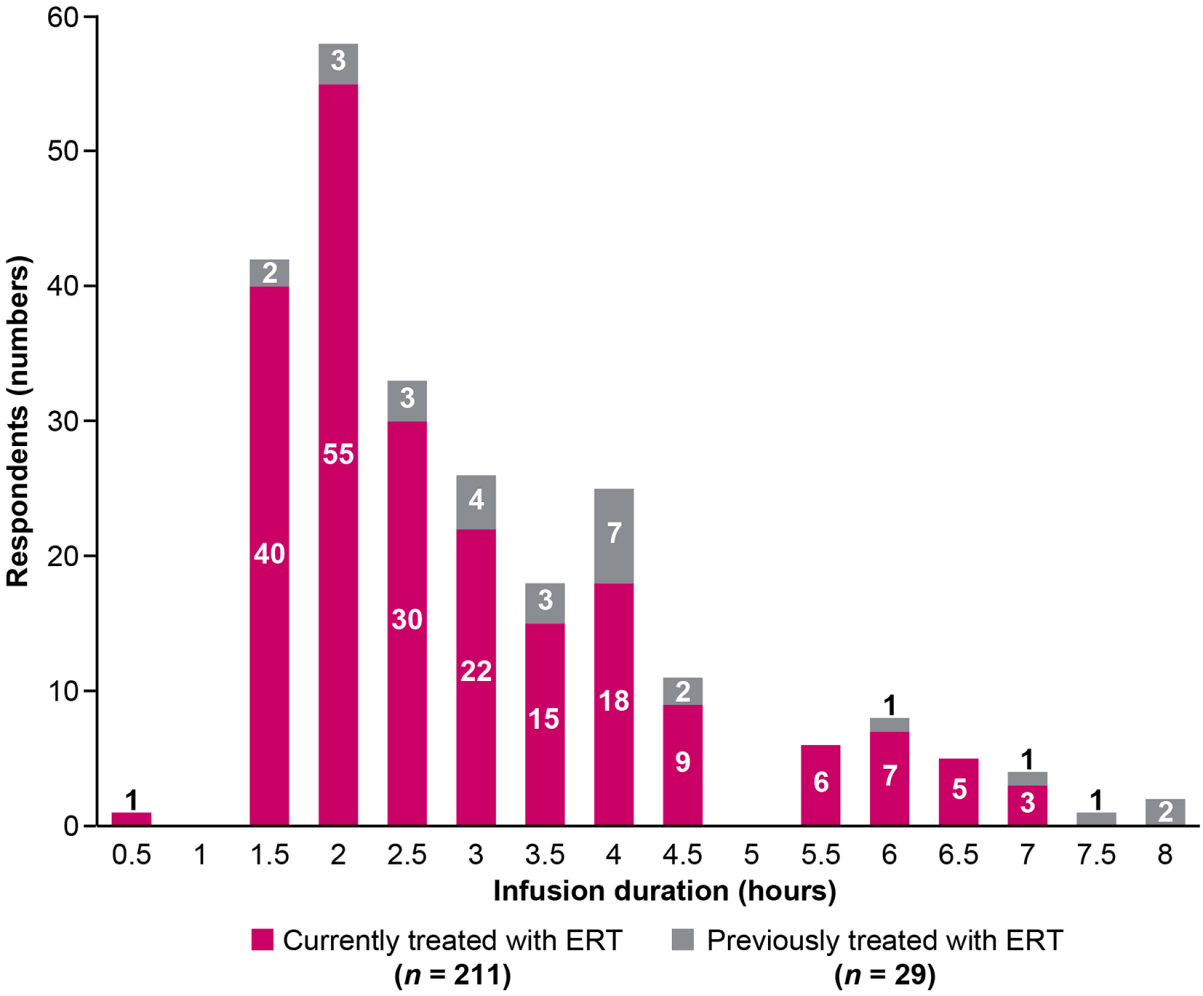
Burden of ERT: Infusion duration reported by respondents currently or previously receiving ERT (*N* = 240). ERT, enzyme replacement therapy

About half of respondents reported that their ERT infusions were administered at home. Infusion duration ranged from 30 min to 8 h (Fig. 4). Approximately 40% (85/211) of respondents currently receiving ERT reported an infusion duration of 3 or more hours; interestingly, more respondents (72%, 21/29) who had previously received ERT, but not currently on ERT, reported a duration of 3 or more hours. Taking medication to manage or prevent IRRs (termed premedication) was reported by 53% (112/211) of respondents who were currently receiving ERT and 66% (19/29) of those who had previously received ERT. Among those who reported taking premedication, 43% (48/112) who were currently receiving ERT and 37% (7/19) who previously received ERT indicated this posed a moderate or significant inconvenience. Among all respondents who currently or previously received ERT, only 36% (87/240) reported they had been tested for ADAs; of those, 30% (26/87) reported they tested positive, 39% (34/87) reported they tested negative, and 31% (27/87) were not able to recall their results. When stratified by gender, 48% (40/84) of males reported having received antibody testing compared with 30% (47/155) of females. Additionally, more females reported not being aware of such a test (16%, 25/155) compared with males (7%, 6/84).

### Symptom worsening between ERT infusions

**Fig. 5:**
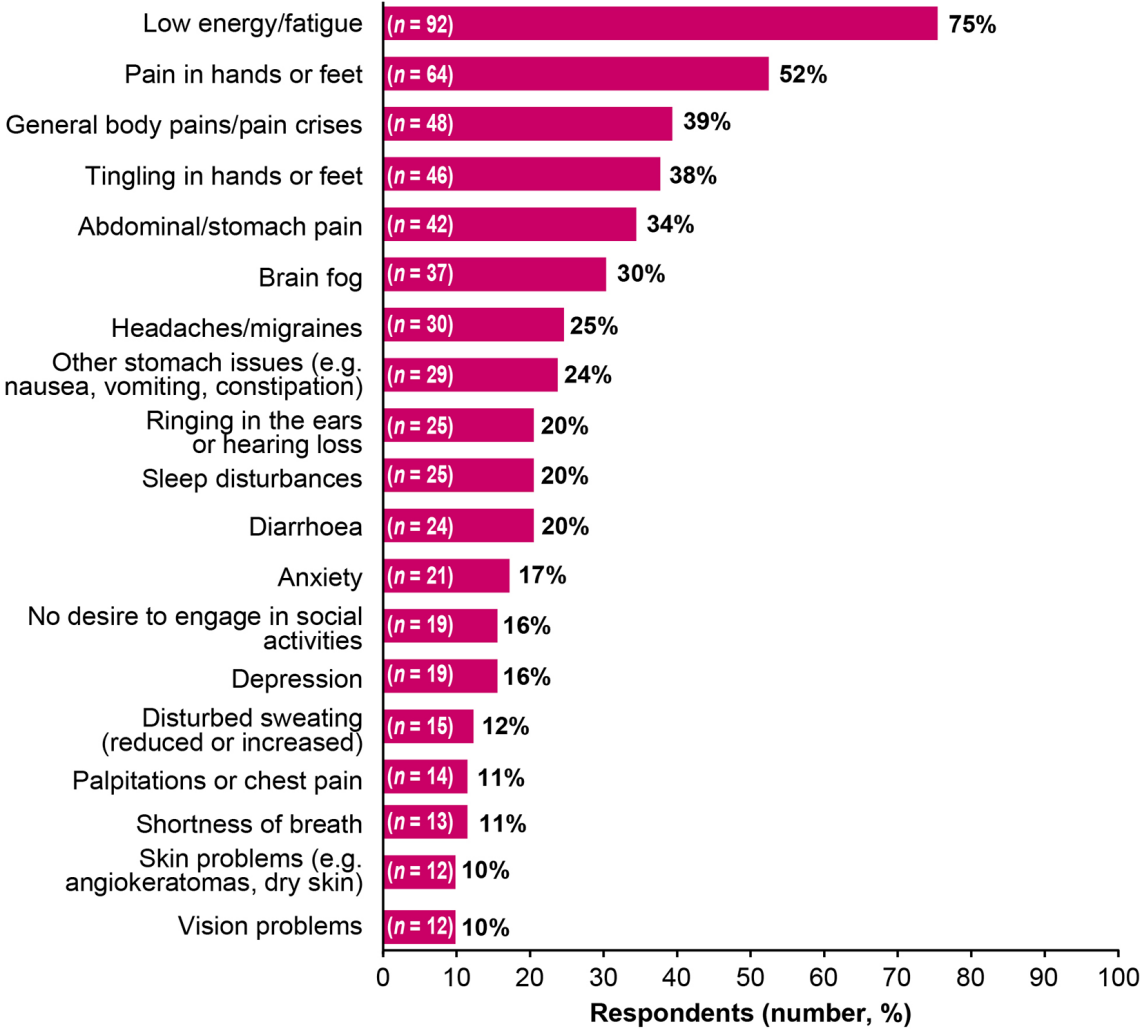
Symptoms reported by respondents who experienced symptom worsening between ERT infusions (*N* = 122). ERT, enzyme replacement therapy

About half of the respondents currently receiving ERT (51%, 108/211) or previously receiving ERT (48%, 14/29) reported temporary symptom worsening between infusions. Among patients who reported symptom worsening between infusions, the most common symptoms reported between infusions were low energy or fatigue (75%, 92/122), pain in hands or feet (52%, 64/122), general body pains or pain crises (39%, 48/122), tingling in hands or feet (38%, 46/122), and abdominal or stomach pain (34%, 42/122) (Fig. 5). Except for low energy or fatigue (male: 59%, 23/39; female: 83%, 68/82), there were no clear differences in symptom worsening when stratified by gender. Respondents most often reported symptom worsening 1 to 2 days (34%, 41/122) or 3 to 4 days (35%, 43/122) before their next infusion was due, and 17% (21/122) reported that symptom worsening could occur at any time between infusions. Only about half (48%, 59/122) reported their symptom worsening to their physician, and of those who reported it, only 41% (24/59) reported that their physician prescribed medication to manage their symptoms or changed their treatment regimen. Among those who previously received ERT and reported their symptom worsening to their physician, all but one reported that their physician did not prescribe medication for symptom management or make any changes to their treatment regimen. Respondents who reported symptom worsening between infusions (*n* = 122) were asked to rate the impact of symptom worsening on QoL on a 5-point scale, wherein 1 denoted no impact, and 5 denoted significant interference with daily activities. The overall median score was 3 (range, 1*–*5); the median score was identical for males and females (range, males: 2–5; females: 1–5), indicating that symptom worsening had a moderate impact on daily activities (score of 3, *n* = 48; score of 4, *n* = 26; score of 5, *n* = 8).

## Discussion

In this analysis, the goal was to capture patients’ perception of their FD, their satisfaction with disease monitoring, and their experiences with ERT treatment. The findings of this survey suggest that despite current standard-of-care treatments, patients with FD continue to experience symptoms, which may negatively impact QoL. This highlights an unmet need for earlier diagnosis and treatment initiation among patients with FD to prevent adverse sequelae that develop over time and demonstrates a need for new therapeutic options. Among respondents who stopped ERT treatment, reported reasons for stopping treatment included the length of infusions, IRRs, and insurance concerns, suggesting barriers to existing ERT treatments that newer treatments should aspire to overcome.

Prior research has highlighted gender differences in FD manifestations, diagnosis, and treatment [26–30]. Several studies have shown that female patients with FD may not be appropriately initiated on ERT or that treatment initiation with ERT may be delayed, despite evidence that females meet the criteria to begin treatment (e.g. they have symptoms such as pain, or they have abnormalities in kidney or heart function) and that females and males have a similar response to treatment [28–30]. In our survey, we observed that female patients comprised a greater proportion of respondents who had never received ERT (90%, 36/40) or had previously received ERT but discontinued (79%, 23/29) compared with respondents who were currently receiving ERT (62%, 132/211). Taken together with these studies, our findings highlight a need to address potential gender disparities to ensure equitable access to recommended treatment for all patients with FD.

To our knowledge, this is the first comprehensive survey focused on the patient-reported burden of ERT infusions, including the use of premedication, as well as the experience of symptom worsening between ERT infusions among adults with FD in the real-world setting. More than half of respondents currently on ERT and nearly two-thirds of respondents previously on ERT reported using premedication; among those who use or have used premedication, 42% (55/131) reported it posed at least a moderate inconvenience. Half of the respondents currently or previously on ERT reported symptom worsening between infusions, and two-thirds of those indicated a moderate impact on QoL and daily activities. These observations raise questions about how ongoing symptoms, and in particular symptoms between treatments, are being managed and highlight a potential opportunity to improve patient care. Despite this symptom burden, only 48% of those with symptom worsening discussed it with their physician. This highlights a need for better communication between patients with FD and their treating physicians, as well as better tools (e.g. questionnaires) to facilitate an open dialogue to identify symptom worsening more effectively between infusions.

Participant responses reveal a deficit and possible disconnect between patient knowledge about disease progression and their perception of disease monitoring or treatment. Although half of the respondents currently receiving treatment for FD reported that they felt their disease was stable and most respondents overall reported satisfaction with how their disease was being monitored, 82% (230 of 280) of all respondents perceived their disease as worsening. These findings suggest that patients with FD may view disease progression as inevitable despite the use of the best available treatment. It also suggests that patients with FD may base their perception on their experience over time rather than on individual laboratory test results indicating stability over smaller periods of time.

In this survey, the monitoring frequency for kidney and heart function reported by most respondents was in accordance with currently published treatment guidelines [5]. However, the re-emergence of pain and GI symptoms between infusions may suggest a need to revisit and revise current guidelines for the management and treatment of patients with FD, particularly related to dosing, timing, and individualized FD treatment recommendations for unique patient subsets. These data suggest that guidelines should include pain-focused PROs as a key aspect of FD management to ensure the patient perspective is more fully considered. Respondents also had limited information of ADA monitoring, which may indicate a need for patient education in this domain. Current guidelines do not offer specific direction on routine ADA monitoring for immunoglobulin G, immunoglobulin E, or neutralizing antibodies to ERTs [16]. Considering that the development of ADAs has been suggested to play a role in symptom worsening between ERT infusions, in addition to their known effect on the safety and efficacy of ERT treatments, guidelines may need to be revised as the effect of ADAs on treatment is clarified [16].

Our survey benefitted from a double-blind, cross-sectional design, administration by a third-party vendor, and a large sample of 280 patients with FD. A limitation of our survey was that the patient cohort may provide an incomplete representation of the global FD patient population. The recruitment method, which primarily relied on patient advocacy groups and physician referrals, may have resulted in an overrepresentation of patients who were actively managed by a physician and were, therefore, better monitored overall. Our survey was conducted online, which meant respondents needed both a computer and internet access; this may have favoured patients with a higher socioeconomic status and may also explain the highly educated population we observed. Because respondents surveyed were patients with FD living in the United States or Canada, they best reflect patients with FD receiving the standard of care and approved treatments in those countries. Most respondents in our survey were women, similar to other surveys of patients with FD [22, 24].

Data collected were self-reported and relied on patient knowledge and experience; these data were not independently verified through chart review. This may impact the accuracy of some data, such as FD type, with approximately a fifth of patients not being aware if they have classic FD. Thus, these results did not seek to explore a potential correlation between FD type and other perceptions and outcomes. For future analyses, patient-reported data should be verified with medical documentation; in particular, it may be of interest to assess the potential disconnect between patients’ understanding of their disease and medical documentation to make impactful comparisons.

FD is a heterogeneous disease and it was outside the scope of this survey to comprehensively assess the impact of each type of symptom or aspect of treatment on patients’ QoL. As such, some questions grouped symptoms by body function affected rather than severity (e.g. tinnitus/hearing loss), limiting the possibility to seek additional correlations between severity of some symptoms and other clinical or baseline characteristics.

## Conclusions

The results of this survey provided valuable insight into the patient experience with FD, including symptoms, symptom frequency, and treatment with ERT and oral chaperone therapy. Our findings highlight the gap between current standard-of-care in disease monitoring and patient perception of disease progression among patients with FD. This indicates that healthcare providers could foster better communication with patients around their illness beliefs and symptoms and provide education to their patients about the connection between monitoring results and disease progression. The information collected from this survey may be helpful for healthcare providers and drug developers seeking to improve the care of patients with FD by addressing unmet needs of high relevance and developing tools that capture more meaningful PROs when evaluating existing or new therapeutic options. A plain language summary of this study is available (See Additional File 2)

### Electronic supplementary material

Below is the link to the electronic supplementary material.

**Additional file 1: Table S1:** Screening and survey questions

**Additional file 2:** Plain language summary (Patient experience with Fabry disease and its management in the real world: a survey of 280 people with Fabry disease)

## Data Availability

We will approve or deny data requests from external parties on a case-by-case basis. Chiesi reserves the right to deny requests for all legally appropriate reasons. Data requests that risk sharing participant-level data or proprietary information will not be approved.

## Abbreviations

ADAs: antidrug antibodies
e.g.: exempli gratia (for example)
ERT: enzyme replacement therapy
FD: Fabry disease
GI: gastrointestinal
IRRs: infusion-related reactions
PROs: patient-reported outcomes
QoL: quality of life
RWE: real-world evidence
