# Supplementary material for: Patient-reported experience with Fabry disease and its management in the real-world setting: results from a double-blind, cross-sectional survey of 280 respondents
Source: Orphanet J Rare Dis. 2024 Apr 11;19:153. doi: 10.1186/s13023-024-03090-4 (PMC11007961; doi:10.1186/s13023-024-03090-4)
Supplement: Supplementary file 1 — Additional file 1: Table S1: Screening and survey questions [file 13023_2024_3090_MOESM3_ESM.docx]

**Additional file 1.**

**Table S1: Screening and Survey Questions**

| **Screening Questions** | | |
| --- | --- | --- |
| S1 | Have you been diagnosed with Fabry Disease by a healthcare professional? | - Yes - No |
| S2 | Please indicate your age (years) | - 1-87 |
| S3 | Are you currently enrolled in a clinical trial for a therapy for Fabry Disease? | - Yes - No |
| S4 | Have you ever been treated with enzyme replacement therapy (ERT)? | S4.1 No, never tried ERT  S4.2 Yes, currently on ERT  S4.3 Yes, received ERT in the past but not currently |
| S5 | What is your current treatment for Fabry disease, if any: | S5.1 Currently receiving oral therapy (Galafold)  S5.2 Not on treatment currently and have not been on treatment in the past  S5.3 Currently on enzyme-replacement therapy (Fabrazyme)  S5.4 Not on any treatment currently but received ERT in the past  S5.5 Not on any treatment currently but received Galafold in the past  S5.6 Other, please specify:  S5.7 Currently on enzyme-replacement therapy (Replagal) |
| **Survey Questions** | | |
| **Demographics and disease history** | | |
| 1 | Please select your gender. | - - Female   - Male   - Transgender Female   - Transgender Male   - Gender variant/Non-conforming   - Prefer not to answer |
| 2 | Are you currently employed? Please select the one that best describes your situation | - Yes, full time - Yes, part time - Student (full-time or part-time) - Stay-at-home household manager - Not employed and not retired - Retired due to disability - Retired not due to disability |
| 3 | Do you have health insurance? Please select all that apply. | - Yes, commercial/private insurance (e.g., insurance provided by your or your partner’s employer) - Yes, Medicare - Yes, Medicaid - Yes, Military (e.g., Tricare) - Yes, Marketplace (offered as part of the Accountable Care Act) - Yes, other insurance type - Not insured |
| 4 | Please indicate your level of education. | - Masters or above - Finished trade school - Finished college - Finished high school or received a GED - Did not finish high school |
| 5 | How long ago were you diagnosed with Fabry Disease? | - Less than one year ago - One to two years ago - 3 to 5 years ago - 6 to 10 years ago - More than 10 years ago |
| 6 | Have you been diagnosed with the classic type (symptoms developing in childhood) of Fabry disease? | - Yes - No - Not sure |
| **Fabry Disease severity and management** | | |
| 7 | How would you characterize the severity of your Fabry disease symptoms? Please choose the one that best applies. | - Mild-moderate symptoms - Bothersome symptoms, Gen - Symptoms are difficult to control |
| 7b | How would you characterize the severity of your Fabry disease in terms of organ damage? Please choose the one that best applies. | - Stable disease without major kidney or heart damage - Kidney or heart function already affected - Worsening kidney or heart function damage |
| 8* | Do you have a history of dialysis or transplant? | - Dialysis (Yes/ No) - Transplant (Yes/ No) - Stroke (Yes/No) |
| 9 | What is the specialty of the primary physician managing your Fabry disease? Please choose one that best applies. | - Primary care/family doctor - Geneticist - Nephrologist (kidney doctor) - Cardiologist (heart doctor) - Neurologist (brain doctor) - No one - Other, please specify: _____ [open end] |
| 10 | How often do you feel that your disease might be worsening even if all the labs and assessments during your doctor(s) visit(s) appear stable? | - Never - Sometimes - Often |
| 11 | Please indicate which of the current symptoms related to your Fabry disease do you currently experience. Please select all that apply. | - Pain in hands or feet - Vision problems - Tingling in hands or feet - Abdominal/stomach pain - General body pains/pain crises - Diarrhea - Other stomach issues (e.g., nausea / Vomiting, constipation) - Low energy/fatigue - Brain fog - Headaches/Migraines - Depression - No desire to engage in social activities - Palpitations or chest pain - Anxiety - Sleep disturbances - Skin problems (e.g., angiokeratomas, dry skin) - Disturbed sweating (reduced or increased) - Ringing in the ears or hearing loss - Shortness of breath - Other (please describe) |
| 12 | Please indicate how well you feel the progression of your disease has been monitored using a scale from 1 to 5 where 1= not monitored well at all and 5=excellent care and monitoring | - 1 = not monitored well at all - 2 = monitored somewhat well - 3 = monitored moderately well - 4 = monitored well - 5 = excellent monitoring |
| 13 | Please indicate how often your kidney or heart function gets assessed by means of lab tests (e.g., creatinine, protein in the urine) or exams (e.g., cardiology visit, ECHO, etc). Choose the option that best describes your situation. | - Kidney – every 3 mo, every 6 mo, every 9 mo, once a year, less frequent than once a year, I’m not sure - Heart - every 3 mo, every 6 mo, every 9 mo, once a year, less frequent than once a year, I’m not sure |
| 14 | If previously discontinued ERT [S4.3 selected], What were the reasons for stopping ERT in the past? Please select all that apply. | - Did not experience any improvement in symptoms - Disease and organ damage kept progressing - Infusions were too lengthy - Experienced infusion reactions - My doctor recommended to stop - Insurance issues - Distance from clinic/infusion center - Switched to oral - Participated in a clinical trial - Other, please specify (open end) |
| 15 | Please indicate how long ago did you start your ERT for Fabry disease.  (Ask if current or prior ERT: S4.2 or S4.3 selected) | - Less than one year ago - One to two years ago - 3 to 5 years ago - 6 to 10 years ago - More than 10 years ago |
| 15a | Please indicate how long ago did you stop your ERT  (Ask if S4.3 selected) | - Less than one year ago - One to two years ago - 3 to 5 years ago - 6 to 10 years ago - More than 10 years ago |
| 16 | Are your ERT infusions currently administered at home?  (Ask if S4.2 selected [“Were your ERT infusions administered at home” ask only if S4.3 selected]) | - Yes - No |
| **Experience with ERT** | | |
| 17 | How long does your ERT infusion currently last (not counting the time for any pre-medications or any wait time or travel time). Please select one.   Ask if S4.2 selected [“How long did your ERT infusion last (not counting the time for any pre-medications or any wait time or travel time)” ask if S4.3 selected] | - 30 minutes - 1 hour 30 minutes - 2 hours - 2 hours 30 minutes - 3 hours - 3 hours 30 minutes - 4 hours - 4 hours 30 minutes - 5 hour 30 minutes - 6 hours - 6 hours 30 minutes - 7 hours - 7hours 30 minutes - 8 hours - 8 hours 30 minutes - 9 hours - 9 hours 30 minutes - 10 hours |
| 18 | Do you currently take medications to prevent infusion-related reactions?   Ask if S4.2 selected Examples …. [“Did you take medications to prevent or manage infusion-related reactions” if S4.3 selected] | - No - Yes, I take 1 medication to prevent infusion reactions - Yes, I take 2 or more medications to prevent infusion reactions - Not sure |
| 19 | [If taking at least 1 medication for infusion reactions in prior question], Please indicate which of following statements best describes your experience with premedications for infusion reactions. Select all that apply. | - Taking premedications for infusion reactions is not an inconvenience for me - Taking premedications for infusion reactions is a moderate inconvenience for me - Taking premedications for infusion reactions is a significant inconvenience for me (e.g., interferes with activities after the infusion, leads to drowsiness, etc.) |
| 20* | Do you experience temporary worsening of symptoms in the days between infusions?   Ask if S4.2 selected [“Do you recall experiencing symptom worsening in the days before your next infusion” ask if S4.3 selected] | - Yes – before most infusions - Yes – before some infusions - No |
| 21 | If you experience(d) temporary symptom worsening between infusions (Yes in previous question 20), please rate from 1 to 5 the impact it has (had) on your quality of life and daily activities (1= no impact and 5=significant interference with my daily activities) | 1 to 5 |
| 22 | If you experience(d) symptom worsening between infusions (Yes in Q20), please describe those symptoms that worsen (select all that apply): | - Pain in hands or feet - Vision problems - Tingling in hands or feet - Abdominal/stomach pain - General body pains/pain crises - Diarrhea - Other stomach issues (e.g., nausea / Vomiting, constipation) - Low energy/fatigue - Brain fog - Headaches/Migraines - Depression - No desire to engage in social activities - Palpitations or chest pain - Anxiety - Sleep disturbances - Skin problems (e.g., angiokeratomas, dry skin) - Disturbed sweating (reduced or increased) - Ringing in the ears or hearing loss - Shortness of breath - Other (please describe) |
| 23 | If you experience(d) symptom worsening between infusions (Yes in Q20), please indicate when those symptoms usually start, on average. | - 1-2 days before the next infusion is due - 3-4 days before the next infusion is due - 4-5 days before the next infusion is due - A week or early before the next infusion - Can be at any time |
| 24 | If you experience(d) symptom worsening between infusions (Yes in Q20), please indicate if you have discussed this with your treating physician. | - No, I never thought to bring this up - No, I did not bring this up because I don’t think the doctor can help me with that - No, I did not not bring this up for another reason - Yes, but the doctor did not make any changes to my treatment - Yes, I have been prescribed medications to manage these symptoms - Yes, the doctor made changes to my treatment |
| 25 | [Ask if S4.2 or S4.3 selected] Have you ever been tested for antibodies against the ERT that you are/were treated with? | - Yes - No - Not sure - Not aware of such a test |
| 26 | If previously tested for anti-drug antibodies (Yes on Q25), what were the results? | - Positive - Negative - Do not recall |
| 27* | [Ask if S4.2 or S4.3 selected] Considering your treatment with an ERT, please indicate how strictly did you observe the infusion schedule of every 2 weeks: | - Less than 50% of the time - Between 50-80% of the time - More than 80% of the time - Not applicable, my physician has recommended a different infusion schedule |
| 28* | [Ask if S4.2 or S4.3 selected] Please indicate the most common reasons that may result in delay or skipping of ERT infusions: | - Symptoms of Fabry disease are very mild - Worsening of Fabry disease symptoms interferes with infusions - Cost of medication - Infusion reactions - No perceived benefit - Too difficult to adapt to dosing schedule - Pre-medication side effects - Interference with school or work - Not applicable, I observe dosing strictly - Vacation, holiday or needed a break - Switching insurance plans or medications required approval that interfered with schedule - Other, specify (free text) |

*Indicates questions for which data is not reported in the manuscript.
