# Supplementary material for: Patient-reported experience with Fabry disease and its management in the real-world setting: results from a double-blind, cross-sectional survey of 280 respondents
Source: Orphanet J Rare Dis. 2024 Apr 11;19:153. doi: 10.1186/s13023-024-03090-4 (PMC11007961; doi:10.1186/s13023-024-03090-4)
Supplement: Supplementary file 2 — Additional file 2: Plain language summary (Patient experience with Fabry disease and its management in the real world: a survey of 280 people with Fabry disease) [file 13023_2024_3090_MOESM4_ESM.pdf]

Patient experience with Fabry disease and its management in the real world: a survey of 280 patients with Fabry disease

Supplemental information to Berry L et al. *Orphanet Journal of Rare Diseases*. 2024.

This is a summary of an article about the patient experience with Fabry disease, including symptoms and treatment, which was published in a medical journal called *Orphanet Journal of Rare Diseases* in 2024.

How to say...

Agalsidase alfa: "AY-gal-suh-days AL-fuh"
Agalsidase beta: "AY-gal-suh-days bay-tuh"
Alpha galactosidase: "al-fuh guh-lak-tow-suh-days"
Fabry: "FAB-ree"

What is Fabry disease?

Fabry disease is a rare, inherited disorder in which patients have a missing, decreased, or nonworking enzyme in their cells. In healthy cells, enzymes are molecules that speed up chemical reactions in the body, such as reactions that create or break down other substances. Patients with Fabry disease have little or no alpha galactosidase A enzyme in their body. As a result, their cells cannot break down large molecules, such as lyso-Gb3. This leads to a build-up of these large molecules in the blood and organs throughout the body. Over time, this causes different symptoms in patients with Fabry disease, such as nerve pain, stomach pain, diarrhoea, fatigue, decreased sweating, hearing loss, and eye problems. Symptoms may get worse over time, and organs such as the heart and kidneys may not function properly. **Fabry disease affects both men and women and is a lifelong disorder**.

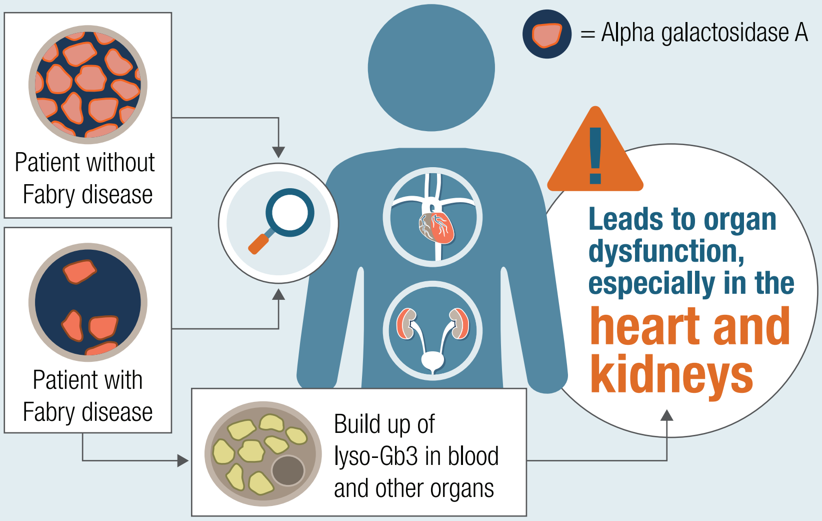

What treatments are available for patients with Fabry disease?

Currently, there are 2 kinds of treatments available for patients with Fabry disease called **enzyme replacement therapy** and **chaperone therapy**. Enzyme replacement therapy replaces the missing enzyme in patients with Fabry disease so their cells can break down lyso-Gb3. At the time of this study, there were 2 medications available for enzyme replacement therapy for patients with Fabry disease: agalsidase alfa and agalsidase beta. A third medication for enzyme replacement therapy, pegunigalsidase alfa-iwxj, was approved in Europe and the United States after this study was completed. All 3 medications are injected directly into the bloodstream by a slow infusion given every 2 weeks.

The other type of treatment, chaperone therapy, works by binding to the nonworking enzyme to restore some of the normal activity. The medication used for chaperone therapy is called migalastat and is taken by mouth every other day. Migalastat can only be used by patients with certain gene changes causing their Fabry disease.

Why did researchers conduct this survey?

There are not many studies that have surveyed patients directly about their disease and treatment experience. This study was conducted to find out how patients with Fabry disease feel about their disease severity, symptoms, monitoring, and treatment.

How was this survey conducted?

Researchers created a 30-minute online survey that had 33 questions. The questions were based on input from healthcare providers who treat patients with genetic diseases and from Fabry disease patient advocacy groups. The survey was completed by patients with Fabry disease who were 18 years or older and could understand English.

Who completed this survey?

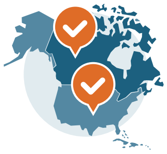

280 patients with Fabry disease in the **US** and **Canada** completed the survey

Treatment history

Currently on any type of treatment:
84% (234 of 280 patients; women: 78% [149 of 191]; men: 95% [84 of 88]; nonconforming: 100% [1 of 1])
Currently on enzyme replacement therapy:
74% (208 patients)

Sex

Women: 68% (191 patients)
Men: 31% (88 patients)
Nonconforming: <1% (1 patient)

Age

Range: 18 to 77 years
Average: 47 years

Work status

Full or part time: 54% (152 of 280 patients; women: 50% [95 of 191]; men: 65% [57 of 88])
Retired with or without disability: 29% (82 patients)
Student: 5% (15 patients)

What did the researchers learn from this survey?

1 What type of doctor primarily managed each patient's Fabry disease?

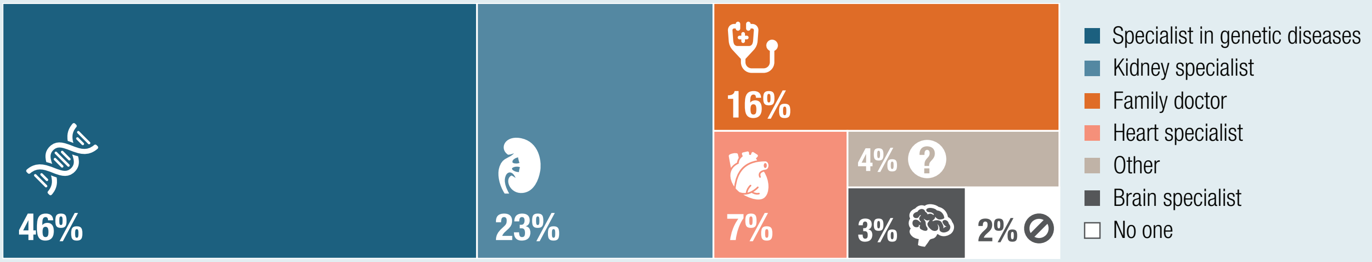

2 What treatment were patients currently receiving?

Among patients receiving treatment for their Fabry disease, 89% (208 patients) were on **enzyme replacement therapy** and 11% (26 patients) were on **chaperone therapy.**

3 What were the most common symptoms patients were currently experiencing?

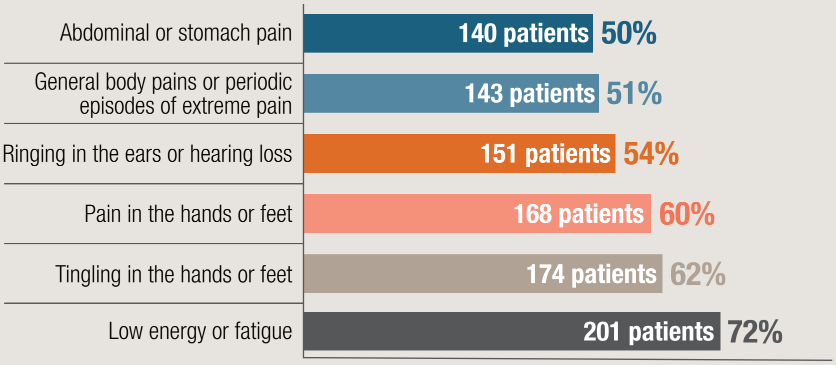

There were no clear differences between men and women.

4 How severe were each patient's Fabry disease symptoms?

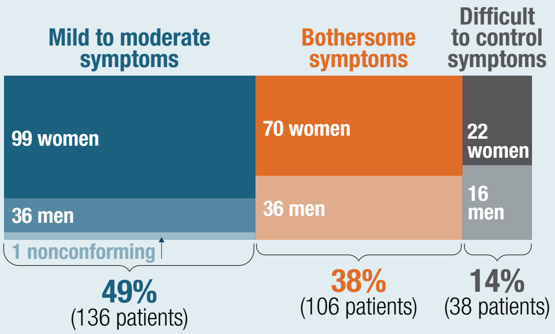

Please note: Percentages do not add up to 100% due to rounding.

5 How often were kidney and heart function monitored using laboratory tests (for example, by measuring creatinine in the blood or protein in the urine) or other examinations/tests (for example, by performing an echocardiogram)?

Most patients were monitored **at least once a year for kidney function** (88%, or 246 patients) **and heart function** (82%, or 230 patients). Women were more likely to be assessed once a year, while more men received testing every 3 months.

6 How did patients feel about their Fabry disease monitoring?

Most patients (82%, or 230 patients) **were happy with how their disease was monitored**, but the same number (82%, or 230 patients) also felt their disease was getting worse even if their monitoring tests suggested it was stable.

7 Did symptoms get worse between infusions?

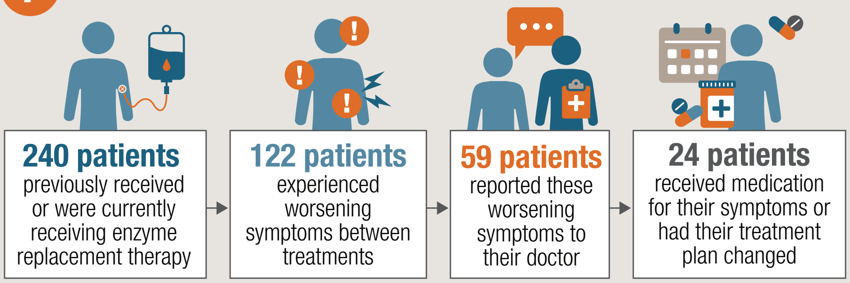

8 How much did symptom worsening impact quality of life?

Overall, most men and women said that symptom worsening had **a moderate impact on their daily life**.

What did this study tell us and why is it important?

This survey gives us important information about the experience of patients with Fabry disease, including their symptoms, monitoring, and treatment. This survey highlights a desire for patients with Fabry disease to better understand the link between how their disease is monitored and how their disease is progressing. To better understand the needs of patients with Fabry disease, doctors should educate their patients on the tests used to monitor their condition and ask specific questions about how they feel about their illness and its progression. This survey may help doctors and drug makers improve the lives of patients affected by Fabry disease by better addressing this community's needs and through providing new treatment options.

More information

This summary is based on the article called "Patient-reported experience with Fabry disease and its management in the real-world setting: results from a double-blind, cross-sectional survey of 280 respondents" which was published in *Orphanet Journal of Rare Diseases* in 2024.

This study was sponsored by Protalix Ltd. and Chiesi. Medical writing support for this summary was provided by Elizabeth Kukielka of Oxford PharmaGenesis Inc., and was funded by Chiesi. The authors thank all the study participants, their families, and the researchers. The original authors of the full article reviewed and approved this summary.
